# COVID-19 in Brazilian indigenous people: a new threat to old problems

**DOI:** 10.1590/0037-8682-0476-2020

**Published:** 2020-08-26

**Authors:** Simone Simionatto, Marcelo Barbosa, Silvana Beutinger Marchioro

**Affiliations:** 1 Universidade Federal de Grande Dourados, Programa de Pós Graduação Strictu Sensu em Ciências da Saúde, Dourados, MS, Brasil.; 2 Universidade Federal da Bahia, Instituto de Ciências da Saúde, Salvador, BA, Brasil.

Dear Editor:

It has been widely reported that coronavirus disease (COVID-19) control requires population surveillance systems to allow the development of effective intervention strategies, especially in vulnerable populations such as indigenous people^1^ ^,^ ^2^. Brazil has 817,000 indigenous people and most of them live in low socioeconomic conditions^3^. There are many challenges to reducing the spread of COVID-19, including cultural issues that can make it difficult to implement control measures, such as social isolation^4^. In addition, limited access to sanitation and potable water, as well as the localization of some communities close to urban areas, increases the vulnerability of indigenous people to COVID-19^4^ ^-^ ^6^.

The Brazilian Indigenous Health Care Subsystem (IHCS) was established by Law No. 9,836 in 1999 to operationalize a model of differentiated care for the indigenous population, which required very differentiated logistics and addressing cultural aspects that need to be incorporated into health care for this population. The National Policy for the Health Attention of Indigenous People (NPHAIP) was enacted in 2002 and brought the elaboration of a work model based on 34 Indigenous Special Sanitary Districts (ISSD) throughout the Brazilian territory. Although there is a specific system for healthcare in the indigenous population, the quality of basic healthcare remains precarious^7^.

 In addition to the difficulty in applying a specific effective health system for this population, the low economic conditions are also an aggravating factor. Despite government efforts to supplement their resources, indigenous people are still among the lowest-income Brazilians^8^. The low socioeconomic conditions, scarcity of indigenous lands, and proximity of their communities to urban areas favor many indigenous people working in urban centers to support their survival. Among the many problems and difficulties faced by the Brazilian indigenous population, this letter seeks to alert the reader to one more problem: the proximity of indigenous reservations to urban centers, which is worsening the situation of this population during the current COVID-19 pandemic.

The first Brazilian indigenous patient with COVID-19 was on April 1^st^ in Amazonas state, and presented 35 days after the first positive case had been reported in the country. According to data from the Indigenous Special Sanitary District (ISSD), which were updated on July 27^th^, there have been 13,728 indigenous people who tested positive for COVID-19 in Brazil, which corresponds to an incidence of 1,716/100,000 inhabitants, with a mortality rate of 1.86%. More than 2/3 of the cases have come from the northern region of Brazil, followed by the northeast, midwest, south, and southeast regions. Ninety-seven deaths have been identified, primarily in the northern region, which currently has the highest mortality rate (39.6/100,000 inhabitants)^9^. 

**FIGURE 1:**
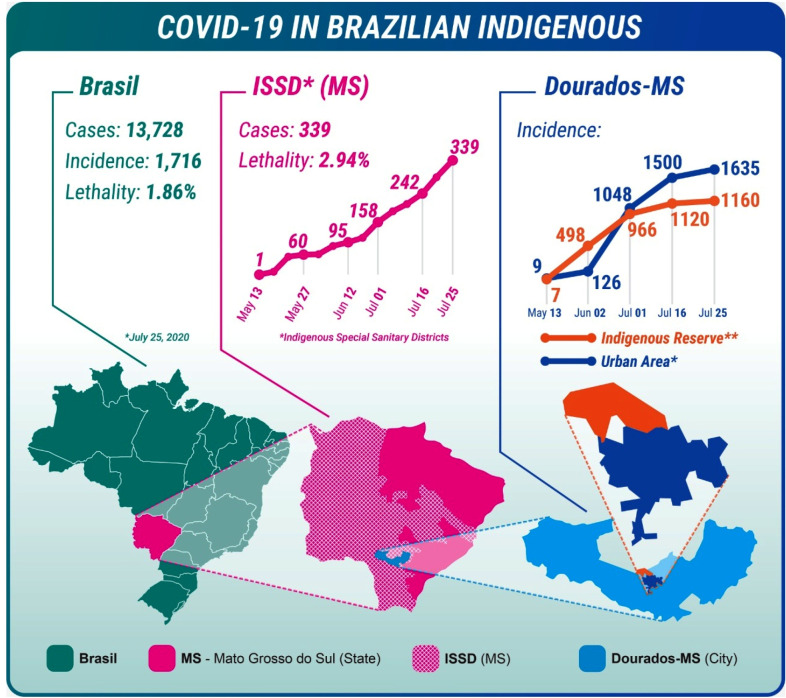
Cases and incidence of COVID-19 in Brazilian indigenous people. Number of COVID-19 cases identified by the Indigenous Special Sanitary District (ISSD) from Mato Grosso do Sul (MS). Geographical location of the Federal Indigenous Reserve of Dourados/MS. COVID-19 incidence in Federal Indigenous Reservation and in urban area of Dourados/MS (incidence: cases/100,000 inhabitants). **Dourados Federal Indigenous Reserve from MS is the second largest urban Indigenous reservation in Brazil.

Mato Grosso do Sul, ISSD, has the largest indigenous population in the country^10^. The first patient who tested positive for COVID-19 in this state was a woman who works in a slaughterhouse, and the case was confirmed on May 13^th^. Her contacts and others indigenous members underwent testing and 70 days later there were 339 cases with a mortality rate of 2.94% (Figure 1). This mortality rate is more than double that for the general population of the state of Mato Grosso do Sul (1.38%). Among the related cases, 174 people living in Dourados Federal Indigenous Reserve (located near an urban area) tested positive, which corresponds to an incidence of 1,160/100,000 inhabitants (Figure 1). To contain the spread of COVID-19 in this reserve, the infected individuals who consented were transferred to an indigenous health facility to maintain social isolation and receive health assistance until they recovered^9^.

The return of the indigenous people from work sites to their communities of origin is a phenomenon that has also contributed to the spread of COVID-19. Migration is a social determinant of indigenous health and has a negative impact in many parts of America^11^. This factor also affects indigenous people living in the Dourados Reserve, as some of these individuals work outside their communities. Moreover, indigenous people usually receive lower wages than their non-indigenous counterparts, and often perform precarious jobs where they experience discrimination and have poor access to benefits and healthcare^12^.

The current epidemiological scenario of COVID-19 reinforces the importance of active surveillance through extensive testing of indigenous people and health professionals. These measures will help identify symptomatic and asymptomatic individuals and thus promote the prompt isolation of patients and their contacts. Therefore, public health policies need to be strengthened based on the needs of these vulnerable and under-served populations, as it remains a recurring issue that is not limited to the current COVID-19 pandemic.
